# A multinational empirical study of perceived cyber barriers to automated vehicles deployment

**DOI:** 10.1038/s41598-023-29018-9

**Published:** 2023-02-01

**Authors:** Shah Khalid Khan, Nirajan Shiwakoti, Peter Stasinopoulos, Matthew Warren

**Affiliations:** 1 School of Engineering, RMIT University, Melbourne, Australia; 2 RMIT University, Melbourne, Australia; 3 University of Johannesburg, Johannesburg, South Africa

**Keywords:** Energy science and technology, Engineering

## Abstract

The digital transformation of Automated Vehicles (AVs) has raised concerns in the cyber realm among prospective AV consumers. However, there is a dearth of empirical research on how cyber obstacles may impact the operation of AVs. To address this knowledge gap, this study examines the six critical cyber impediments (data privacy, AV connectivity, ITS infrastructure, lack of cybersecurity regulations, AV cybersecurity understanding, and AV cyber-insurance) that influence the deployment of AVs. The impact of gender, age, income level, and individual AV and cybersecurity knowledge on these obstacles are statistically assessed using a sample of 2061 adults from the United States, the United Kingdom, New Zealand, and Australia. The research revealed intriguing empirical findings on all cyber barriers in the form of a trichotomy: participants' education level, understanding of AVs, and cybersecurity knowledge. As education levels increase, the significance of a cyber barrier to AV deployment decreases; however, as AV comprehension and cybersecurity knowledge increase, the perception of a cyber barrier becomes significantly more important. In addition, the study demonstrates differences in perceptions of cyber barriers and AV deployments based on gender, age, income, and geographic location. This study's findings on cyber barriers and AV deployment have implications for academia and industry.

## Introduction

The deployment of Automated Vehicles (AVs) will serve as the primary impetus for the delivery of innovative solutions for a safer, more efficient, and sustainable Intelligent Transportation System (ITS). AV's essential characteristic is its ability to enable ubiquitous connectivity, particularly real-time operation, which refers to data flow in the form of information and controls among ITS stakeholders^1^. AVs can operate in one of the six automation stages, ranging from Level 0 to Level 5, with Level 5 denoting complete automation^2^.

The digital transformation of AVs, specifically the operation of Levels 4–5, has raised concerns in the cyber realm among prospective AV consumers. For instance, the anxiety that Personally Identifiable Information (PII) generated during the operation of AV may be mishandled. PII includes user information, i.e., geographic information, private conversations in the vehicle, travel patterns, biometric authentication, and vehicle specifications^3^. Similarly, the integration of AI-enabled 6G and low-latency 5G into Vehicle-to-Everything (V2X) communication will usher in a new age of smart mobility by allowing seamless availability, 3D holographic display, and augmented reality^4^. However, its failure is also a cause for worry. Maeng, et al.^5^ found that customers see communication failure (connectivity) as the most significant hazard to the safe functioning of AV. Similarly, ITS infrastructure, such as machine-readable road signs, smart roadway markings, or sensors, is required to function AV^6^. However, the pace of ITS infrastructure is significantly slower than that of AV technology, which is also a cause for anxiety. Infrastructure was identified as one of the significant positive predictors of the urgency level of AV deployment in Taiwan^7^.

The digital revolution and the incorporation of multiple stakeholder groups in AV operations resulted in new legal challenges. The primary difficulty is reducing criminal behaviour in both the physical and digital realms, which requires the application of AV cybersecurity policies and, more importantly, the legislative framework to ensure adequate cybersecurity compliance. Moreover, another potential barrier to AV deployment is the public's lack of understanding and awareness of the technology's hazards and implications^8^. Trust in any newly produced technology is intrinsically tied to education and an individual's grasp of that technology. Furthermore, the lack of clarity about product liability is a significant cause of worry among prospective AV purchasers. For example, it is unclear who is accountable for responsibility claims arising from AV Level 4–5 failures and crashes. Would the automaker, software vendor, or connectivity service provider be held liable, or is it the customer's fault for failing to follow the onboard computer's instructions^9^?

Similarly, the primary characteristic of AV is ubiquitous connectivity (flow of data, controls, and directives) among ITS stakeholders. For instance, AVs are anticipated to generate 2500 gigabytes per hour from 200 sensors^10^. Given the unprecedented growth of data volumes, AV-data governance (data owners, data stewards, and data custodians) can be challenging. Establishing standards and compliance requirements for exchanging real-time data between stakeholders, levelling the playing field for all stakeholders, protecting consumer rights and privileges (privacy), and mitigating criminal behaviour represent the most significant obstacles. One potential strategy could be the timely integration of a flexible, open format data management architecture like “Lakehouse^11^”, which could help with AV data management, improve data governance and security, and provide deeper insights into the data.

In recent years, concerns about AV operations in cyberspace have emerged and attracted the attention of both academia and industry. Despite not being included in traditional acceptance models, research shows that cyber-related concerns are significant predictors of AV adoption as well as deployment^12^. However, there are still substantial knowledge gaps in this field. First, efforts to comprehend the public's acceptance of AVs are still limited, and their psychological determinants are largely unknown^13^. Second, the obstacles that have emerged as a result of cyber-related realms are investigated in a limited dimension with a focus on adoption only; primarily as perceived risk, either as an antecedent for trust in adoption or as an antecedent for adoption directly^14^. The emphasis is predominantly on assessing the acceptance of AVs in terms of safety, privacy, security, and performance^15^. The literature lacks an assessment of novel avenues for AV deployment that are prevalent among the general public, such as vehicle internet connectivity or ITS infrastructure, self-driving vehicle insurance, or a lack of potential cybersecurity regulation^16^. The authors^14,17^ have stressed the importance of further research into how cybersecurity influences the willingness to use AVs.

In addition, perceptions of cyber obstacles may vary by age, gender, income, educational background, and even cyber-related expertise. For instance, women tend to be more worried about safety than men^12^, and the effect of security awareness on perceived anxiety is contingent on the form of security awareness: psychological interest and attention vs. cognitive comprehension and practical application^18^. Likewise, recent studies on AV deployment in relation to cyber challenges tend to focus on specific locations, so their findings may not be applicable to other parts of the world where AV technologies are rapidly developing. The contextual influence and focus of privacy concerns differ between individualistic and collectivistic countries^19^. Thus, it remains unexplored how consumers' worries about AV cyber obstacles vary in the context of various countries with distinct cultural traditions. Nonetheless, there is a scarcity of empirical research to thoroughly examine relationships among demographic variables, cyber barriers, and AV deployment.

To address these knowledge gaps, this study shifts the emphasis from AV adoption to AV operation. It is the first to examine the six dimensions of cyber barriers and measure their impact on AV deployment using a massive dataset from a diverse population. By examining the influence of different demographic categories on AV adoption, this study contributes to understanding the perception of AV cyber barriers: data privacy, AV connectivity, ITS infrastructure, lack of cybersecurity regulations, AVs cybersecurity understanding, and AV cyber-insurance. In doing so, we add a comprehensive and fine-grained analysis of public and cyber obstacles, AV acceptability, and policies to the literature on AV deployment. Nonetheless, the study has conducted a comprehensive literature review to provide a state-of-the-art understanding of the challenges and obstacles that AVs may face in terms of user acceptance as a result of the emergence of the cyber domain.

## Dimensions of the AV cyber barriers

The following sub-sections provide an overview of the scope, importance, and ramifications of the challenges raised by internet connectivity in AV operations. The six dimensions are derived systematically through a meta-exploratory literature review. Over 170 articles and documents were selected to track the evolution of AV technology and the emergence of cyber barriers that impact the deployment of AVs. The authors' previous literature review work on cyber-attacks on next-generation cars and anticipated readiness^1^ and the model for cybersecurity assessment of AVs^16^ contributed towards the identification of six AV cyber impediments.

### Data privacy

The data privacy concerns are threefold. First, at the consumer level, it includes demographic details such as vehicle information, driver's licence, real-time location, travel patterns, and video and in-car communication of the AV users^1^. The authors of Atmaca, et al.^20^ claimed that location data might be utilised to correlate with identity. Other temporal data may be utilised to infer additional personal data, such as home or work locations, age, occupation, behavioural characteristics, habits, and social ties. Second, proximity espionage: the monitoring of AV sensors (laser, radar, camera, lidar) intrudes significantly on the privacy of nearby people/assets. For instance, 360-degree surveillance of non-verbal communication, i.e., bodily movement (bio-signalling) data, identifies a person^21^. Similarly, sensor eavesdropping concerns prompted the Chinese military to ban Tesla vehicles from their camps^22^. The third is the privacy of the vendor's intellectual property, such as a person pleading guilty in the United States to a Tesla ransomware plot for infringing intellectual constrictions^23^.

Most studies of AV’s public perceptions have focused on demographic variables such as gender, age, and income for their independent variables. A survey carried out in the US found that people who are less likely to adopt AVs are those who are more concerned with their privacy when using them^24^. The study on individuals of various genders and ages reveals diverse concerns about data privacy when using AVs^25^. The findings of a multi-nation survey indicate that respondents from more developed nations (as measured by lower accident rates, better levels of education, and higher wealth) were less comfortable with their car-sharing data^26^.

In contrast, several studies reveal that privacy is of relatively low concern to respondents regardless of their geographical location^27^. The reasons could be that AVs have yet to hit the road or a lack of awareness about AV-data flow. Therefore, to investigate the impact of demographic factors: gender, age, income level, geographic locations, and individual AV and cyber-knowledge on the data privacy of AV consumers, it is hypothesised that:

**H1a: **The perception of data privacy as a barrier to the operation of AVs has a significant difference across gender.

**H1b: **The perception of data privacy as a barrier to the operation of AVs has a significant difference among individuals of different age groups.

**H1c: **The perception of data privacy as a barrier to the operation of AVs has a significant difference among individuals of different income levels.

**H1d: **The perception of data privacy as a barrier to the operation of AVs has a significant difference among individuals of different education levels.

**H1e: **The perception of data privacy as a barrier to the operation of AVs has a significant difference among individuals who understand AVs.

**H1f: **The perception of data privacy as a barrier to the operation of AVs has a significant difference among individuals with cybersecurity knowledge.

**H1g: **The perception of data privacy as a barrier to the operation of AVs has a significant difference among individuals in the United States, the United Kingdom, New Zealand, and Australia.

### AV connectivity

AVs are distinguished by their pervasive connectivity with ITS infrastructure and other vehicles in order to enable autonomous movements and shared-route management^1^. Next-generation wireless communication technologies, including 5G and 6G, would enable seamless mobility and 3D holographic displays in AV operations. Khan et al.^1^ presented an AVs connectivity framework with a flowchart to depict interfaces for potential cyber hazards, providing a systematic understanding of AVs connectivity and analysing potential cyber risks on different interfaces. When new links are established between AV and ITS infrastructure, the potential hazard scope is expanded via intermediate nodes^15^.

Given the public's interest in this subject, assessing whether it could pose a challenge to the widespread use of AVs is essential. Internet connectivity research indicates an effect on customer adoption. For instance, the authors^28^ discovered that internet banking failures reduce user engagement. The sensitive nature of vehicle-mounted data services (such as driving route and positioning monitoring) further exacerbates the connectivity concerns. Nonetheless, the risk of connectivity is regarded as a crucial factor in the adoption of AVs^29^. Hence, to examine the impact of demographic factors on the connectivity of AVs, it is hypothesised that:

**H2a: **The perception of AV connectivity as a barrier to the operation of AVs has a significant difference across gender.

**H2b: **The perception of AV connectivity as a barrier to the operation of AVs has a significant difference among individuals of different age groups.

**H2c: **The perception of AV connectivity as a barrier to the operation of AVs has a significant difference among individuals of different income levels.

**H2d: **The perception of AV connectivity as a barrier to the operation of AVs has a significant difference among individuals of different education levels.

**H2e: **The perception of AV connectivity as a barrier to the operation of AVs has a significant difference among individuals who understand AVs.

**H2f: **The perception of AV connectivity as a barrier to the operation of AVs has a significant difference among individuals with cybersecurity knowledge.

**H2g: **The perception of AV connectivity as a barrier to the operation of AVs has a significant difference among individuals in the United States, the United Kingdom, New Zealand, and Australia.

### ITS infrastructure

ITS provides innovative solutions for more coordinated and efficient transportation infrastructure use. The key feature is the use of advanced information and communication technologies to collect data on road conditions and enable AVs to operate in real time. ITS infrastructure can be classified into two categories: (i) road-side entities such as machine-readable road signs (e.g., UV QR overlay or PCM-modulated light signals), smart road marking, sensors, (ii) service providers, including V2X candidates and spectrum sharing etc. Determining compliance requirements, contractual, legal, industry standards, and regulatory requirements, as well as privacy demands, comes immediately after infrastructure has been established^30^. Furthermore, ITS infrastructure would influence the urban environment^31^, such as lowering congestion due to fewer accidents, reducing the number of vehicles on the road due to increasing ridesharing, or requiring less parking space. Public acceptability is a prerequisite for adopting new technology regarding technological demand and infrastructural investments ^32^. The lack of suitable ITS infrastructure between adjacent geographic locations can impede AV mobility and its adoption. Therefore, a uniform ITS infrastructure is required for the successful deployment of AVs. This includes, but is not limited to, the installation of sensors, cameras, and other technologies that enable V2X communication, the development of uniform regulations and standards for AV operation, the establishment of a nationally consistent platform for AV data governance, and the provision of skilled human resources to govern the operations of AVs.

Currently, the pace of ITS infrastructure is significantly slower than AV technologies, especially resilient cyber infrastructure. According to a study conducted in the United Kingdom, public concern regarding the infrastructure's ability to support automated driving is growing^33^. The study^34^ indicate that different levels of concern exist regarding recognising traffic signs and markings. Hereafter, to examine the impact of demographic factors on the ITS infrastructure, it is hypothesised that:

**H3a: **The perception of ITS infrastructure as a barrier to the operation of AVs has a significant difference across gender.

**H3b: **The perception of ITS infrastructure as a barrier to the operation of AVs has a significant difference among individuals of different age groups.

**H3c: **The perception of ITS infrastructure as a barrier to the operation of AVs has a significant difference among individuals of different income levels.

**H3d: **The perception of ITS infrastructure as a barrier to the operation of AVs has a significant difference among individuals of different education levels.

**H3e: **The perception of ITS infrastructure as a barrier to the operation of AVs has a significant difference among individuals who understand AVs.

**H3f: **The perception of ITS infrastructure as a barrier to the operation of AVs has a significant difference among individuals with cybersecurity knowledge.

**H3g: **The perception of ITS infrastructure as a barrier to the operation of AVs has a significant difference among individuals in the United States, the United Kingdom, New Zealand, and Australia.

### AVs cybersecurity regulation

The digital revolution, the rise of new economic prospects, and the inclusion of diverse stakeholder groups in AV operations have all resulted in new legal challenges. The key challenge is mitigating criminal behaviour in both the physical and digital worlds, which necessitates the implementation of AV cybersecurity policies and, more critically, the cybersecurity regulatory framework to assure acceptable cybersecurity compliance^35^. The regulatory framework would impact product (AV) liability, the insurance domain, vehicle ownership, travel pattern privacy, driver licencing and infrastructure regimes, ITS architects, and motor vehicle-related crimes. This has led to ambiguity for AV customers regarding data accessibility, ownership, usage, business model, and privacy, as well as the legislative frameworks that govern these channels both domestically and internationally. Moreover, regulatory approaches to the definitions of safety differ among nations, although AV technology is employed globally.

Existing legal frameworks are inadequate in the face of disruptive AV technology. Although the global adoption of cybersecurity regulations is increasing, there is a lack of AV-specific cybersecurity regulations that do not hinder their adaptation. The lack of regulation is a cause for concern for the public. A study conducted by Underwood^36^ with 217 transportation experts shows that regulations are among the most challenging impediments to the deployment of AVs. The authors^37^ demonstrated that establishing standards and laws for AVs is important in promoting their increased adoption. Considering that AVs collect extensive data about users and their travel patterns, the degree to which regulations safeguard user privacy will determine how widely AVs are adopted^38^. The authors^7^ found that regulation was one of the significant negative predictors of the urgency level of AV development in Taiwan. Therefore, to examine the impact of demographic factors on the lack of AV cybersecurity regulation, it is hypothesised that:

**H4a: **The perception of the lack of AV cybersecurity regulation as a barrier to the operation of AVs has a significant difference across gender.

**H4b: **The perception of the lack of AV cybersecurity regulation as a barrier to the operation of AVs has a significant difference among individuals of different age groups.

**H4c: **The perception of the lack of AV cybersecurity regulation as a barrier to the operation of AVs has a significant difference among individuals of different income levels.

**H4d: **The perception of the lack of AV cybersecurity regulation as a barrier to the operation of AVs has a significant difference among individuals of different education levels.

**H4e: **The perception of the lack of AV cybersecurity regulation as a barrier to the operation of AVs has a significant difference among individuals who understand AVs.

**H4f: **The perception of the lack of AV cybersecurity regulation as a barrier to the operation of AVs has a significant difference among individuals with cybersecurity knowledge.

**H4g: **The perception of the lack of AV cybersecurity regulation as a barrier to the adoption of AVs has a significant difference among individuals in the United States, the United Kingdom, New Zealand, and Australia.

### AVs cybersecurity comprehension

A significant impediment to effective barrier prevention is the public’s inadequate understanding and awareness of risk factors^8^. The literature reveals that individuals with a high level of education have a more positive attitude towards AVs than those with less education^27^. Likewise, AVs cybersecurity comprehension is a critical tool for protecting AVs against cyber hazards^16^. Education is inextricably linked to trust in any newly developed technology and the ability of individuals to appropriately categorise cybersecurity risks varies. Therefore, to examine the impact of demographic factors and the lack of education on the cybersecurity of AVs, it is hypothesised that:

**H5a: **The perception of the lack of AV cybersecurity comprehension as a barrier to the operation of AVs has a significant difference across gender.

**H5b: **The perception of the lack of AV cybersecurity comprehension as a barrier to the operation of AVs has a significant difference among individuals of different age groups.

**H5c: **The perception of the lack of AV cybersecurity comprehension as a barrier to the operation of AVs has a significant difference among individuals of different income levels.

**H5d: **The perception of the lack of AV cybersecurity comprehension as a barrier to the operation of AVs has a significant difference among individuals of different education levels.

**H5e: **The perception of the lack of AV cybersecurity comprehension as a barrier to the operation of AVs has a significant difference among individuals who understand AVs.

**H5f: **The perception of the lack of AV cybersecurity comprehension as a barrier to the operation of AVs has a significant difference among individuals with cybersecurity knowledge.

**H5g: **The perception of the lack of AV cybersecurity comprehension as a barrier to the operation of AVs has a significant difference among individuals in the United States, the United Kingdom, New Zealand, and Australia.

### AVs cyber insurance

The lack of clarity regarding product liability is a major source of concern among potential AV consumers. It is unclear, for instance, who is liable for the liability claims resulting from AV Level 4–5 crashes and failures. Would responsibility be transferred to the manufacturer, the ITS service provider, the software vendor, or the customer for failing to follow on-vehicle computer instructions? Since the insurance industry is based on actuarial risk assessment, potential cyberattacks have increased uncertainty in this domain, which may be too high to impose on the industry without a significant premium increase. If cybersecurity insurance is not required, increased premiums will likely discourage the purchase of such coverage. However, if cybersecurity insurance is required for vehicle owners, this could limit the adoption of this technology^39^. Quantitative evidence indicates that liability is one of the most challenging barriers to deploying AVs^7,36^. Therefore, to examine the impact of demographic factors on AV's cyber insurance, it is hypothesised that:

**H6a: **The perception of the lack of AV cyber insurance as a barrier to the operation of AVs has a significant difference across gender.

**H6b: **The perception of the lack of AV cyber insurance as a barrier to the operation of AVs has a significant difference among individuals of different age groups.

**H6c:**The perception of the lack of AV cyber insurance as a barrier to the operation of AVs has a significant difference among individuals of different income levels.

**H6d:**The perception of the lack of AV cyber insurance as a barrier to the operation of AVs has a significant difference among individuals of different education levels.

**H6e:**The perception of the lack of AV cyber insurance as a barrier to the operation of AVs has a significant difference among individuals who understand AVs.

**H6f:**The perception of the lack of AV cyber insurance as a barrier to the operation of AVs has a significant difference among individuals with cybersecurity knowledge.

**H6g:**The perception of the lack of AV cyber insurance as a barrier to the operation of AVs has a significant difference among individuals in the United States, the United Kingdom, New Zealand, and Australia.

## Methodology

This section describes the questionnaire and constructs specifics, participant profiles, and data analysis methodologies.

### Questionnaire survey

The study design is a framework for data collecting and analyzing and linking the collected data to the research objectives. The Society of Automotive Engineers (SAE) defines six levels of driving automation. Level 0: No automation Level 1: Assisted Driving Automation; Level 2: Partial Automation; Level 3: Conditional Automation; Level 4: High Automation; Level 5: Full Automation^2^. The scope of research is focused on cars or on-road motor vehicles that have functionality levels 4 or 5. The questionnaire developed for this study consists of three sections. The consent block and participant information sheet are in the first section, which also contains a brief project description and an overview of AVs operation.

The second section includes driving-related information, AV knowledge, and demographic information for respondents (detailed in the next section). The third section of the survey questioned each barrier resulting from AV operations in cyberspace, emphasising AV adoption research. These dimensions were developed after a comprehensive literature review using the notion of perceived AV cyber-based operation. Subsequently, it was refined based on expert evaluation, which included consultation with three human factors specialists, four undergraduate students, four PhD researchers (interdisciplinary), three cybersecurity professionals, and four automotive industry experts. The constructs proposed in this work will make an important contribution to the literature due to the scarcity of parameters at the level specific to perceived AV cyber impediments. On a five-point Likert scale ranging from "not at all important" (= 1) to "extremely important" (= 5), respondents were asked to rank the significance of each cyber dimension as a barrier to the operation of self-driving vehicles. Furthermore, all methods were carried out in accordance with relevant guidelines and regulations, and the Science, Technology, Engineering, and Mathematics College of Human Ethics Advisory Network (after a series of revisions) approved this study's ethics application (Reference Number 25065). Participants who were at least 18 years old were included in the study, and informed consent was obtained from all the participants. A pilot test indicated that the questionnaire took roughly 7–9 min to complete.

### Participants

The survey questionnaire was administered online by Qualtrics, a credible and trustworthy third-party research service provider used by numerous researchers and organisations all over the globe to conduct their studies. Qualtrics was informed of the requirement as well as the inclusion and exclusion criteria, such as the speeder checker to complete the survey, the mandatory answer to all questions, CAPTCHA, etc., via written contract. Qualtrics maintains a database of willing participants who are compensated in accordance with the terms negotiated with them. Participants who qualified for the study were forwarded to the next page of the questionnaire, and a soft launch of 200 respondents served to validate the pilot test.

In full launch, a total of 2062 respondents over the age of 18 from four countries (the United States, the United Kingdom, New Zealand, and Australia) are considered, with an equal distribution (and verification via GeoIP). The reasons for data collection from these countries are threefold: citizens of these nations are largely digitally savvy and wary of cyber-related incidents^40^; (ii) the trend for AV adoption in these nations is marked by the presence of exciting AV projects with substantial investments^41–43^; and (iii) majority of resident in these nations spend a significant amount of time on the road. Furthermore, data is collected from each region in order to have a nationally representative sampling–as per the census region breakdown per country, as follows: **United States**: Northeast, Midwest, South, West; **Australia**: New South Wales, South Australia, Victoria, Territory/Australian Capital Territory, Queensland, Tasmania/Northern, and Western Australia; **New Zealand**: Auckland, Lower North Island, South Island, Upper North Island; and **United Kingdom**: Northern England, Southern England, Mid England, Greater London, Wales, Northern Ireland, and Scotland. Table 1 summarises the driving-related information and demographic characteristics of the respondents.

**Table 1:** Demographics and characteristics of participants.

Category	Variable	Frequency	Percentage
Gender	Male	946	45.88%
	Female	1097	53.20%
	Others	12	0.58%
	Prefer not to say	7	0.34%
Age	18–24	296	14.35%
	25–35	384	18.62%
	36–45	374	18.14%
	46–55	346	16.78%
	55–65	325	15.76%
	65 and above	337	16.34%
Country	Australia	524	25.41%
	United Kingdom	502	24.35%
	New Zealand	511	24.78%
	US	525	25.46%
Australia	New South Wales	141	26.91%
	Queensland	118	22.52%
	Victoria	150	28.63%
	Western Australia	49	9.35%
	South Australia	38	7.25%
	Tasmania / Northern Territory / Australian Capital Territory	28	5.34%
United Kingdom	Northern England (Northwest, North East, Yorkshire & the Humber	125	24.90%
	Mid England (West Midlands, East Midlands & East of England)	134	26.69%
	Southern England (Southwest & South East)	117	23.31%
	Wales	27	5.38%
	Greater London	67	13.35%
	Scotland	17	3.39%
	Northern Ireland	15	2.99%
New Zealand	Auckland	196	38.36%
	Upper North Island	94	18.40%
	South Island	107	20.94%
	Lower North Island	114	22.31%
US	Northeast	83	15.81%
	Midwest	112	21.33%
	South	214	40.76%
	West	116	22.10%
Income	0–$25,000	345	16.73%
	25,001–$50,000	535	25.95%
	$50,001–$75,000	397	19.25%
	$75,001–$100,000	293	14.21%
	$100,001–$125,000	163	7.90%
	$125,001–$150,000	128	6.21%
	$150,001–$175,000	77	3.73%
	$175,001–$200,000	53	2.57%
	$200,001–$225,000	32	1.55%
	$225,001 +	39	1.89%
Education	High School, Certificates/Diploma/Equivalent	1039	50.39%
	Undergraduate: Bachelor’s degree/ Equivalent	776	37.63%
	Master's degree	199	9.65%
	Doctoral degree	48	2.33%
Heard of self-driving/driverless vehicle	Yes	1866	90.49%
	No	147	7.13%
	Not sure	49	2.38%
Understanding self-driving/driverless vehicles?(Follow-up question based on previous question)	Not well at all	375	19.58%
	slightly well	693	36.24%
	Moderately well	566	29.56%
	Very well	196	10.29%
	Extremely well	83	4.33%
Have you heard of cybercrime/cybersecurity?	Yes	1699	82.40%
	No	264	12.80%
	Not sure	99	4.80%
How well do you understand cybercrime/cybersecurity?(Follow-up question based on previous question)	Not well at all	251	13.96%
	Slightly well	681	37.88%
	Moderately well	565	31.42%
	Very well	218	12.12%
	Extremely well	83	4.62%

Females account for 53% of respondents, while males make up 45%, with ages ranging from 18–24 (14%), 25–25 (18%), 36–45 (18%), 46–55 (16%), 55–65 (15%), and 65 + (16%). The most common range of annual income was 25,001–50,000 US$ (25%) followed by 50–0001-75,000 US$ (19%) and 75,001–10,000US$ (14%). A high school diploma was held by the majority of respondents (50%), followed by a bachelor's degree (37%), then a master's degree (9%), and a doctoral degree (2%) as well. The 90% of respondents had heard of AVs, and 52% had driven a vehicle with automated features such as cruise control or self-parking, with an understanding of AVs operation ranging from "slightly well (36%)", "moderately well (29%)", and "Not at all (19%)", and only 11% had taken a ride on an automated vehicle. Moreover, 82% of respondents were familiar with the term's cybersecurity and cybercrime, with 37% understanding the concepts "slightly well," 31% "moderately well," and 4% "extremely well".

### Data analysis

The survey data were statistically analysed using the Mann–Whitney U test and the Kruskal–Wallis H test, intended to investigate the significance of differences among grouping variables as determined by preference scales^27^. The Mann–Whitney U test is a non-parametric statistical test that is used to compare the medians of two groups when the responses (data) for the test (dependent) variable are ordinal and to determine whether the rankings of the two groups differ significantly^44^. The Kruskal–Wallis test is a Mann–Whitney U test extension that evaluates the statistical significance of more than two groups. Statisticians widely acknowledge that the *p*-value cannot be interpreted in isolation but must be considered in the context of certain design and substantive application features, such as sample size and meaningful effect size^45,46^. Because the sample size in our study is quite large (i.e., 2061), *p*-value less than 0.05 are acceptable for reporting significance of hypothesis decisions. The findings were analysed using IBM SPSS Statistics Version 28.0.1.1 software (15).

## Results

To assess the perception of cyber obstacles in the operation of AVs, the Mann–Whitney U test and Kruskal–Wallis test are used. For demographics with two groups, such as gender (male and female), the Mann–Whitney U test is used. However, Kruskal-test Wallis's is being used for more than two grouping variables. Prior to applying these tests, the following assumptions were verified: i) dependent variables are measured ordinally; ii) independent variables consist of two groups in the Mann–Whitney U test and more than two groups in the case of the Kruskal Walli's test; and iii) each observation is independent. For each obstacle's evaluation, tests were conducted independently. The significance-based summary of the hypothesis is presented in Table 2**.**

**Table 2:** Significance-based summary of the hypothesis.

Hypothesis	Asymp. Sig	Decision	Hypothesis	Asymp. Sig	Decision
H1a	0.03	Supported	H4d	0.022	Supported
H2a	0.49	Not Supported	H5d	0.012	Supported
H3a	0.76	Not Supported	H6d	0.031	Supported
H4a	0.26	Not Supported	H1e	< 0.001	Supported
H5a	0.11	Not Supported	H2e	0.002	Supported
H6a	0.45	Not Supported	H3e	0.031	Supported
H1b	0.006	Supported	H4e	0.012	Supported
H2b	< 0.001	Supported	H5e	0.003	Supported
H3b	< 0.001	Supported	H6e	0.291	Not Supported
H4b	< 0.001	Supported	H1f	< 0.001	Supported
H5b	< 0.001	Supported	H2f	< 0.001	Supported
H6b	< 0.001	Supported	H3f	0.014	Supported
H1c	0.135	Not Supported	H4f	< 0.001	Supported
H2c	0.391	Not Supported	H5f	< 0.001	Supported
H3c	0.016	Supported	H6f	0.032	Supported
H4c	0.214	Not Supported	H1g	0.319	Not Supported
H5c	0.074	Not Supported	H2g	0.223	Not Supported
H6c	0.098	Not Supported	H3g	0.446	Not Supported
H1d	0.005	Supported	H4g	0.100	Not Supported
H2d	0.026	Supported	H5g	0.065	Not Supported
H3d	0.006	Supported	H6g	0.455	Not Supported

Table 1 (of supplementary material) shows the Mann–Whitney U test results to determine the statistical significance of the gender effect on barriers to AV adoption. The findings revealed a statistically significant difference between male and female respondents in data privacy as an impediment to adoption (Mann–Whitney U = 490,266; *p* = 0.03). Data privacy was ranked higher in female respondents (mean rank = 1047) than in male respondents (mean rank = 992); hence H1a is accepted. However, because the difference in other barriers was not statistically significant, i.e., H2a, H3a, H4a, H5a, and H6a were not accepted.

Table 2 (of supplementary material) shows the Kruskal–Wallis H results to determine the statistical significance of the age effect on barriers to AV adoption. The findings revealed a statistically significant difference in all of the obstacles to adoption by age group. Hence H1b, H2b, H3b, H4b, H5b, and H6b were accepted. Interestingly, a trend shows an increase in the perception of a barrier to the adoption of AV technology with an increase in the age group, as shown by mean ranks.

The results of the Kruskal–Wallis H test to determine the statistical significance of the income effect on barriers to AV adoption are presented in Table 3 (of supplementary material). The data demonstrated a statistically insignificant difference in the majority of cyber barriers perceptions, except ITS infrastructure (Kruskal–Wallis H = 20.38, *p* = 0.016). Participants with high incomes rated the ITS infrastructure barrier as less essential; hence, H1c was supported. H1c, H3c, H4c, H5c, and H6 were not accepted.

The impact of education level on barriers to AV adoption was analysed using the Kruskal–Wallis H test, the results of which are shown in Table 4 (of supplementary material). The findings revealed a statistically significant difference among educational level and each of the barriers to AV adoption. Thus, H1e, H2e, H3e, H4e, H5e and H6e were accepted. It is worth noting that there is a trend that shows a decrease in the perception of a barrier to the adoption of AV technology as education level increases as indicated by mean ranks values.

Table 5 (of supplementary material) presents the findings of the Kruskal–Wallis H test to determine the statistical significance of the understanding of AVs in relation to cyber barriers on AV adoption. Participants' perceptions of cyber impediments to the adoption of AVs differed significantly, except AVs cyber insurance. Subsequently, H1f, H2f, H3f, H4f, and H5f were accepted, except and H6f. Surprisingly, those who understood AVs "extremely well" rated the majority of cyber obstacles as extremely important to the adoption of AVs.

Table 6 (of supplementary material) shows the results of the Kruskal–Wallis H test to determine the statistical significance of cybersecurity knowledge level in relation to cyber barriers on AV adoption. Participants' perceptions of cyber impediments to AV adoption varied significantly, according to the data. As a result, H1g, H2g, H3g, H4g, H5g, and H6g were all accepted. Those who knew cybersecurity "extremely well" rated cyber obstacles as extremely important to AV adoption.

Moreover, Table 7 (of supplementary material) shows the results of the Kruskal–Wallis H test, which was used to determine the statistical significance of the individuals belonging to the United States, the United Kingdom, New Zealand, and Australia in relation to cyber barriers to AV adoption. Empirical evidence shows that there is no statistical significance in the differences in participants' perceptions of cyber impediments to the adoption of AVs. Subsequently, H1h, H2h, H3h, H4h, H5h, and H6h were not accepted.

## Discussion

AV's digital transformation requires an adoption analysis based on cyber barriers. The notion of cyber impediments when using AVs has been highlighted in the literature as a critical worry while riding AVs. However, existing AV literature has failed to assess the various dimensions of these obstacles to deployment. This study offers a unique perspective by delving into the characteristics of perceived cyber obstacles associated with the operation of AVs.

Cyber impediments can manifest in a variety of ways, each of which may have a distinct effect on the public's acceptance of AVs. Consumers have built an imagined perception of AVs based on information from various sources. It is essential to understand these perspectives since they will form the basis of future choices despite consumers' lack of direct experience with AVs. For example, it will be extremely difficult to persuade individuals to use these technologies when they become available if their perception of the barriers to using AVs is negative based on the information currently available. Therefore, we argue that analysing the perception of AV cyber obstacles is crucial, as these factors will have a significant impact on the future acceptability of AVs based on their operation.

### Implications for theory

The present work makes a substantial addition to theoretical knowledge by elucidating the major cyber challenges associated with operating self-driving vehicles. Based on the state-of-the-art literature and our understanding of the phenomenon, we proposed six main dimensions of obstacles caused by the operation of AVs in cyberspace: data privacy, AVs connectivity, ITS infrastructure, lack of cybersecurity regulations, AVs cybersecurity understanding, and AV cyber-insurance. Therefore, the most important contribution of this study is to shed light on how different demographic variables are associated with the six areas of public concern that serve as dependent variables using a massive dataset from diverse geographic areas and profiles.

The AV literature is updated with empirical evidence that, among cyber barriers to AV deployment, the perception of data privacy is the only one that differs by gender. As an impediment to the acceptance of AVs, female respondents regarded data privacy as more significant than male respondents. The results are consistent with previous research. For instance, a study conducted by Sørum, et al. ^47^ for the synthesis of data protection legislation revealed that women were less likely than men to provide more sensitive information but more willing to disclose their birthdate, television viewing history, and shopping history.

The study's findings revealed a statistically significant difference in perception of all six barriers to AV operation by age group. With increasing age, it demonstrated an increased perception of a barrier. This supports Eby, et al. ^48^ finding that older adults reflect a cautious approach to vehicle automation. Also, older adults may comprehend technology differently than younger generations^49,50^.

The study's empirical results showed that the effect of education level on barriers to AV deployment is statistically significant. It determines that the perception of a barrier to AV technology decreases as the education level grows. According to Rogers, et al. ^51^, early adopters often have a higher academic background. Individuals with higher levels of education tend to be more optimistic about the potential of AVs than those with lower levels of education^27^.

Similarly, the study finds that the familiarity of AVs operating on AV deployment varies significantly depending on data privacy, AV connectivity, the absence of cybersecurity regulation, and AV cybersecurity comprehension. Those with an in-depth understanding of AV identified the majority of cyber obstacles as essential to the broad use of AVs. The literature has constantly shown that comprehending AVs is crucial to their acceptance^16^, and our research gives empirical support for this notion. Likewise, the research illustrates the impact of cybersecurity awareness levels concerning cyber obstacles on AV adoption. Those with "extensive" knowledge of cybersecurity regarded cyber barriers as extremely important to AV operation.

The study's results demonstrate the significance of the income factor in AV deployment of cyber- barriers. Only ITS infrastructure perceptions vary by socioeconomic status among the six cyber obstacles. Participants with high incomes rated the ITS infrastructure barrier as less essential. This supports the dichotomous findings in the literature regarding the effect of income on the perception of AV acceptance. Most studies showed no relationship between income and AV adoption, and only a few publications established a correlation between income and adoption ^52^. Nevertheless, the study's findings demonstrate no statistically significant differences in the perceptions of cyber barriers to AVs deployment among participants from the United States, the United Kingdom, New Zealand, and Australia. The lack of a multinational survey assessing cyber barriers to AV deployment makes this an important contribution to the literature on AVs.

### Implications for practice and policy

It is essential to emphasise that AV's cyber impediments are based on perceptual factors based on sentiments derived from many sources of information that are often difficult to influence. Consequently, many of the implications discussed here concern how ITS stakeholders (telecommunications service providers, road operators, automobile manufacturers, and policymakers) can influence these perceptions to promote the widespread use of AVs. Decision-makers' engagement in risk and innovation is multifaceted ^53^. They may play a pivotal role in minimising the perception of all six cyber barriers highlighted in the study.

The primary requirement is ensuring the data privacy of AV consumers. As an impediment to the acceptance of AVs, female respondents regarded data privacy as more important than male respondents. This highlights that surveillance and data exploitation affect us all. However, by learning about the unique experiences and challenges faced by women, decision-makers can better understand how patriarchy and systems of oppression function. To target 49.58% of the world's female population for AV adoption early, automakers and ITS service providers must balance data accessibility constraints with consumers' data privacy concerns^9^.

Automated driving technologies have the potential to improve the mobility of the elderly; however, the study's empirical findings reveal that the perception of obstacles increases with age. Older people are not a homogeneous group; they represent a range of experiences and needs that must be taken into account when introducing new technology^54^. ITS stakeholders must address this concern; one possible solution is to educate this cohort and hold AV simulation-based and real-world test-ride events. There is evidence that older individuals are more motivated to accept new technologies when they regard the technology as beneficial^55^.

Similarly, the research presented an intriguing empirical finding in the form of a trichotomy, which consisted of the participants' education level, AV comprehension, and cybersecurity knowledge. First, the perception of a cyber barrier to the deployment of AV technology does diminish as the education level rises. Second, as individuals become more familiar with AVs, these obstacles to deployment become more critical. Thirdly, a comprehensive understanding of cybersecurity makes cyber obstacles to AV operation more pertinent. This highlights the complexity and dynamic nature of cyber obstacles for AVs and challenges the notion that participants with a high level of education are early adopters. Additionally, it necessitates measures to address the concerns of those with a high level of education, a thorough understanding of AVs, and a deep understanding of cybersecurity. Furthermore, the study reveals that there is no difference in perception of cyber barriers between individuals with different income levels and geographic locations, such as Australia, the United States, the United Kingdom, and New Zealand. This demonstrates that, at least in these nations, decision-makers can take comparable measures to reduce AV cyber obstacles.

Nonetheless, to ensure that all cohorts of society are early adopters of AV, the most crucial aspect for practitioners is to establish a comprehensive set of integrated cyber obstacle mitigation strategies^1^. A multifaceted approach must be employed to boost the public acceptability of AVs and alleviate the cyber worries around AV technology. This includes "mitigating cyber-concerns by design," i.e., limiting personal data acquisition to what is essential while guaranteeing complete functioning. In addition, anonymize the AV user's identity before disclosing driving behaviour or other pertinent data. Importantly, there is a need for an AVs regulatory framework in cyberspace. Decision-makers should focus on establishing a regulatory framework based on automaker innovation and sharing risks in eliminating negative externalities caused by underinvestment and knowledge asymmetries in cybersecurity. Likewise, there is opportunity to capitalise on massive AV-generated data in AV operations^56^, and safeguard consumer data.

## Conclusion and future direction

The speed and scope of AV automation are significantly influenced by public opinion, and we contend that a comprehensive understanding of this opinion is essential for the widespread deployment of AVs. However, there is a dearth of empirical research on how cyber obstacles may impact the operation of AVs. Therefore, to get ahead of the curve, this study examines the six critical cyber impediments that influence the deployment of AVs: data privacy, AVs connectivity, ITS infrastructure, lack of cybersecurity regulations, AVs cybersecurity understanding, and AVs cyber-insurance. The impact of gender, age, income level, and individual AV and cybersecurity knowledge on these obstacles is statistically assessed using a sample of 2061 adults from the United States, the United Kingdom, New Zealand, and Australia.

The statistical evidence demonstrates that different age groups perceive the six barriers to AV deployment differently, with the significance of each barrier increasing with age. Only the perception of data privacy differs by gender; female respondents perceive data privacy as a more important barrier to the deployment of AVs than male respondents. There was no statistically significant difference between genders in perception of other cyber barriers. The research uncovered an interesting empirical finding regarding the cyber obstacles in the form of a trichotomy: the participants' level of education, their comprehension of AVs' operation, and their cybersecurity expertise. The impact of a cyber barrier on AV deployment declines as education levels rise, but when AV understanding and cybersecurity expertise rise, the perception of a cyber barrier becomes much more essential.

In addition, the perception of cyber barriers to AV adoption was consistent across various income levels and geographic locations, including Australia, the United States, the United Kingdom, and New Zealand. This suggests that regardless of income or location, decision-makers in these nations can take identical steps to eliminate AV cyber barriers. This research may be useful for ITS stakeholders as it reveals the primary areas of promise and anxiety among the global public due to the integration of the cyber realm in AV operations.

Though the empirical synthesis of assessing cyber-impediments in AVs' operations is emerging, cyber dimensions are proliferating, necessitating additional research. The study, for example, assesses AV cybersecurity regulation, but more research is needed to determine the impact of compliance and regulatory rules on the collection, processing, and storage of AV consumer PII data. As the use of AVs increases and more data is collected and shared, it is critical that consumers understand and have control over how their PII is collected, processed, and stored as per compliance rules like General Data Protection Regulation (GDPR). Although GDPR has improved consumers' privacy rights, there are still a few loopholes. For example, data brokers continue to collect and sell consumer information, and the online advertising industry is rife with potential abuses^57^.

## Supplementary Information

Supplementary Information.

## Data Availability

The datasets generated and analysed during the current study are not publicly available due to pending university approval for sharing raw data, but they are available upon reasonable request from the corresponding author.
